# Remodeling of the Host Cell Plasma Membrane by HIV-1 Nef and Vpu: A Strategy to Ensure Viral Fitness and Persistence

**DOI:** 10.3390/v8030067

**Published:** 2016-03-03

**Authors:** Scott M. Sugden, Mariana G. Bego, Tram N.Q. Pham, Éric A. Cohen

**Affiliations:** 1 Laboratory of Human Retrovirology, Institut de Recherches Cliniques de Montréal (IRCM), 110 Pine Avenue West, Montreal, QC H2W 1R7, Canada; scott.sugden@ircm.qc.ca (S.M.S.); mariana.bego@ircm.qc.ca (M.G.B.); tram.pham@ircm.qc.ca (T.N.Q.P.); 2 Department of Microbiology, Infectiology and Immunology, Université de Montréal, C.P. 6128, Succursale Centre-ville, Montréal, QC H3C 3J7, Canada

**Keywords:** HIV-1, Vpu, Nef, host cell surface proteins, replication, immune evasion

## Abstract

The plasma membrane protects the cell from its surroundings and regulates cellular communication, homing, and metabolism. Not surprisingly, the composition of this membrane is highly controlled through the vesicular trafficking of proteins to and from the cell surface. As intracellular pathogens, most viruses exploit the host plasma membrane to promote viral replication while avoiding immune detection. This is particularly true for the enveloped human immunodeficiency virus (HIV), which assembles and obtains its lipid shell directly at the plasma membrane. HIV-1 encodes two proteins, negative factor (Nef) and viral protein U (Vpu), which function primarily by altering the quantity and localization of cell surface molecules to increase virus fitness despite host antiviral immune responses. These proteins are expressed at different stages in the HIV-1 life cycle and employ a variety of mechanisms to target both unique and redundant surface proteins, including the viral receptor CD4, host restriction factors, immunoreceptors, homing molecules, tetraspanins and membrane transporters. In this review, we discuss recent progress in the study of the Nef and Vpu targeting of host membrane proteins with an emphasis on how remodeling of the cell membrane allows HIV-1 to avoid host antiviral immune responses leading to the establishment of systemic and persistent infection.

## 1. Introduction

The plasma membrane (PM) of metazoan cells is by definition the interface between the intracellular and extracellular world. This glycoprotein-rich lipid bilayer controls the intracellular environment by regulating the flux of molecules in and out of the cell. Furthermore, the PM plays a dominant role in cellular communication via cell-to-cell contacts and soluble messenger molecules, resulting in changes in cellular activation states and metabolism, cellular proliferation, and homing of cells throughout the body. This mode of cellular communication is particularly important for immune responses during viral infection. Indeed, a large number of host innate and adaptive antiviral immune responses rely on cell-to-cell contacts to coordinate immune defenses as well as to identify and eliminate infected cells.

To maintain proper homeostasis, the protein and lipid components of the PM are finely controlled through a complex energy-consuming system of vesicular transport to and from the cell surface. In addition, the lateral location of molecules within the membrane is tightly controlled by directed vesicular transport and molecular microdomains restricting the free movement of molecules to confined regions on the PM. Membrane tuning is highly dynamic and responsive, allowing for precise control of energy/waste flux, ionic balance, and cell-to-cell attachment, amongst other effects. As expected, dysregulation of membrane homeostasis plays a role in a wide range of human diseases [1,2].

Human immunodeficiency virus type I (HIV-1) is a retrovirus of the *Lentivirus* genus that causes a chronic and persistent infection in humans. The virus infects primarily CD4+ T cells as well as macrophages and co-opts numerous cellular machineries to achieve optimal replication and dissemination to different tissues and organs. This ultimately leads to Acquired Immune Deficiency Syndrome (AIDS), a condition characterized by loss of CD4+ T cells, profound immunodeficiency, and susceptibility to serious opportunistic infections [3]. HIV infection is defined by several stages of progression. Acute infection is the earliest stage and is characterized by a high level of systemic viral multiplication and a massive, irreparable loss of gut-associated CD4+ T cells. The development of immune responses against HIV-1 occurs after the first few weeks of infection and leads to some control of viral replication, primarily through virus-specific CD8+ cytotoxic T lymphocyte (CTL) responses, as reflected by the establishment of stable set point viremia three to six months after infection. Acute infection is followed by a chronic infection stage that typically lasts eight to ten years. This clinically asymptomatic stage, which is characterized by persistent HIV replication, systemic immune activation, inflammation, and the gradual depletion of CD4+ T cell, leads to the development of AIDS in the absence of antiretroviral therapeutic interventions. Recent studies of transmitted viruses (termed transmitter/founder (T/F) viruses) [4,5] have demonstrated the extraordinary evolutionary fitness required to achieve efficient mucosal transmission. T/F virions must undergo initial propagation at the port of entry despite early immune responses, and subsequently expand to draining lymph nodes to establish a systemic infection [6,7]. It is becoming increasingly clear that the first few weeks following HIV-1 infection are extremely dynamic and represent a critical window in which HIV-1 either establishes a systemic and persistent infection, which includes the establishment of latent viral reservoirs impervious to current antiretroviral drug regimens, or is stifled by insufficient viral expansion and spread, leading to failed infection [8].

Given the important roles of the PM in cellular metabolism, homing, communication, and especially immune surveillance, it is not surprising that HIV-1 has evolved specialized proteins that manipulate the organization and composition of the PM of infected cells to avoid host antiviral immune responses and establish persistent systemic infection. Indeed, HIV-1 encodes two accessory proteins, negative factor (Nef) protein and viral protein U (Vpu), which function primarily by altering the quantity and quality of cell surface molecules to increase viral fitness despite host antiviral immune responses. Expressed at different stages in the HIV-1 life cycle, Nef and Vpu employ a variety of mechanisms to target both unique and redundant host cell surface proteins, including the CD4 viral receptor, restriction factors, immunoreceptors, homing molecules, tetraspanins and membrane transporters. 

In this review, we discuss the roles of HIV-1 Nef and Vpu in the modification of the cell membrane composition and organization with an emphasis on how these alterations increase viral fitness by promoting HIV-1 dissemination while preventing immune detection of infected cells.

## 2. Negative Factor (Nef) Protein

Nef is a 27–35 kDa protein produced early in the HIV life cycle from a multiply-spliced transcript [9]. Although Nef is not essential for virus replication *in vitro*, humans infected with Nef-deficient HIV-1 display greatly reduced viral titers [10,11], maintain higher CD4+ T cell counts and generally do not progress to AIDS [11]. Analogous outcomes are observed when macaques are infected with Nef-deficient simian immunodeficiency virus (SIV) [12].

**Figure 1:**
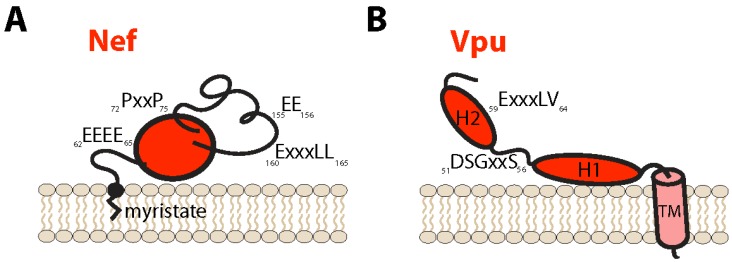
Structure of human immunodeficiency virus type I (HIV-1) negative factor (Nef) protein and viral protein U (Vpu). (**A**) HIV-1 Nef is a soluble protein found associated with cellular membranes through a myristate moiety added post-translationally to a glycine residue at amino acid (aa) position 2. Nef is composed of a compact globular core region (aa 58–149, 181–206) a flexible N-terminus (aa 1–57) and a C-terminal loop (aa 150–180). Conserved motifs of Nef include an adaptor protein (AP)-1/2-binding di-leucine sorting signal and a coatomer subunit beta (β-COP)-binding di-acidic motif both found within the C-terminal loop (aa 160-ExxxLL-165 and 155-EE-156, respectively), a Src family kinase (SFK)-binding polyproline domain (aa 72-PxxP-75) found within the core, and a phosphofurin acid cluster sorting proteins (PACS)-binding acidic cluster (aa 62-EEEE-65) in the N-terminal region; (**B**) HIV-1 Vpu is a transmembrane (TM) protein comprised of a 4 aa luminal tail, a single α-helical TM domain and a cytosolic domain that includes a flexible linker region and two α-helices (H1 and H2) flanking a conserved dual serine motif (aa 51-DSGxxS-56). The dual serine motif of Vpu is readily phosphorylated by casein kinase-2 (CK-II), promoting the recruitment of the beta-transducin repeats-containing proteins (β-TrCP) component of the Skp, Cullin, F-box-containing (SCF)^β−TrCP^ E3 ubiquitin ligase complex. An AP-1/2-interacting sorting motif (aa 59-ExxxLV-64) is found in the second α-helix of the cytoplasmic domain of most group M Vpus. Numbering of aa is based on the Vpu and Nef proteins from the prototypical pNL4-3 molecular clone of HIV-1.

A portion of Nef is found associated with the PM via a myristic acid moiety added post-translationally at the N-terminus of the protein [9]. Nuclear magnetic resonance (NMR) studies suggest that Nef is composed of a globular core region (amino acids (aa) 58–149, 181–206), a flexible N-terminal region (aa 1–57), and a C-terminal loop (aa 150–180) (Figure 1A). An aspartate moiety at aa 123 is required for homo-dimerization and has been shown to be essential for most Nef functions [13]. Nef acts primarily as a linker molecule by connecting its protein targets to components of the endosomal/vesicular machinery, resulting in their trafficking away from the cell surface towards the *trans*-Golgi network (TGN) or lysosomes. Functionally important regions of Nef include the aa 160-ExxxLL-165 motif which interacts with members of the adaptor protein family (AP-1 and AP-2), an acidic cluster domain (aa 62-EEEE-65) required for binding to phosphofurin acid cluster sorting proteins (PACS-1 and -2) [14,15], a polyproline domain (aa 72-PxxP-75) required for binding to Src family kinases (SFKs) [16], and a di-acidic motif (aa 155-EE-156) which interacts with the coatomer subunit beta (β-COP) [17]. It is noteworthy that AP-1/2, PACS-1/2 and COP proteins all serve important roles in vesicular trafficking.

## 3. Viral Protein U (Vpu)

Vpu is a 16–17 kDa type I membrane protein composed typically of a 4 aa luminal domain of unknown function, a 23 aa transmembrane (TM) domain, and a 56 aa cytosolic tail made up of a membrane-proximal flexible hinge region followed by two α-helices bracketing an important linker region containing a well-conserved aa 51-DSGxxS-56 motif (Figure 1B). Vpu is produced from a singly-spliced transcript produced late during the virus life cycle which also encodes the Env glycoprotein precursor in an alternate reading frame [18]. The cellular localization of Vpu varies depending on the HIV-1 clade. For instance, Vpu from clade B of the pandemic group M HIV-1 localizes primarily within the vesicular system, particularly in the TGN, while clade C Vpu is found both in intracellular compartments and at the PM [19]. The DSGxxS motif of Vpu is readily phosphorylated by the cellular casein kinase-2 (CK-II) at serine residues 52 and 56 (S52,56) [20], resulting in recruitment of the beta-transducin repeats-containing proteins 1 or 2 (β-TrCP1/2) subunit of the Skp, Cullin, F-box-containing (SCF)^β−TrCP1/2^ E3 ubiquitin ligase complex [21]. Through this interaction, Vpu is classically thought to function primarily as a liaison between its cellular targets and ubiquitin ligase machinery.

The *vpu* gene is present in HIV-1 and its precursor, chimpanzee-infecting simian immunodeficiency virus (SIVcpz), but is absent in the related but less virulent HIV-2 or its precursor, the SIV infecting sooty mangabeys (SIVsmm) [22,23]. Similar to Nef, monkeys infected with Vpu-defective hybrid HIV-SIV viruses (SHIV) have 10 to 100-fold lower blood virus titers and generally maintain normal CD4+ T cell counts compared to animals infected with isogenic Vpu-competent SHIV [24]. In addition, Vpu has been shown to be important for the initial HIV-1 expansion during acute infection in humanized mouse models [25,26]. 

## 4. Downregulation of the CD4 Viral Receptor: Prevention of Superinfection, Enhancement of Viral Release and Protection from Antibody-Dependent Cell-Mediated Cytotoxicity (ADCC)

The CD4 molecule expressed on CD4+ T cells and macrophages is the primary receptor for HIV-1 entry. Binding of the luminal subunit (gp120) of the trimeric HIV Env glycoprotein complex to CD4 results in conformational changes in gp120, inducing the exposure of previously occluded domains needed to bind the HIV co-receptors CCR5 and/or CXCR4. Subsequent molecular events lead to HIV-1 envelope fusion with the cell membrane of target cells and ultimately viral entry. Both HIV-1 Nef and Vpu target CD4 to reduce its expression at the cell surface [27,28]. CD4 downregulation prevents superinfection of cells, a condition that may induce premature cell death [29] and also hypothetically ensures that infectious virions are not lost to redundant infection events. In addition, CD4 downregulation promotes viral egress by preventing the binding of newly synthesized gp120 protein to CD4 within the vesicular system prior to virion assembly [30] or at the cell surface where CD4 can act as a “tether”, preventing the release of infectious particles [31].

Recently, CD4/gp120 interactions have also been linked to anti-HIV ADCC activity. ADCC is a mechanism whereby natural killer (NK) cells, monocytes and neutrophils [32] bind the fragment crystallizable (Fc) region of antibodies (Abs) coating the surface of antigen-expressing cells via specialized Fc receptors (FcγRIIIa; CD16) to promote their cytolytic activity, ultimately leading to the killing of antibody-coated target cells. ADCC is antigen specific, with Abs against certain epitopes generating greater ADCC responses than others [33]. ADCC has been shown to play a role in the anti-HIV immune response, with NK cells appearing to be the major effectors of anti-HIV activity [34,35].

Increased interest was drawn towards the role of ADCC in the control of HIV-1 infection when the presence of Abs generated against ADCC-favored epitopes was correlated with protective immunity in the recent RV144 vaccine trial [36]. The protection-associated Abs generated by this vaccination regimen were mainly of the immunoglobulin (Ig)G1 and IgG3 isotypes and targeted various locations of gp120, including the C1 and V1/V2 regions [36,37,38,39]. Interestingly, the non-neutralizing A32 antibody (Ab), a potent mediator of ADCC isolated from patient blood over 20 years ago, is known to recognize a conformational epitope that overlaps the C1 and C4 regions of gp120 that is exposed after CD4/gp120 binding (CD4-induced; CD4i) [38,40]. Importantly, many of the ADCC-promoting Abs that conferred some level of protection in the RV144 trial are A32-like, targeting similar CD4i epitopes [39]. While these results suggest that the presence of Abs targeting CD4i regions of gp120 can contribute to protection against HIV-1 transmission, ADCC-competent Abs that preferentially bind gp120 in the CD4-bound conformation are also present in sera from non-vaccinated HIV-1-infected individuals [38,41,42]. The fact that ADCC responses alone are insufficient to clear infection shows that HIV-1 has most likely developed escape mechanisms to avoid exposure of CD4i epitopes at the surface of infected cells.

Recent studies have indeed revealed that accumulation of CD4 at the cell surface, a process counteracted by Nef and Vpu, sensitizes infected cells to ADCC by promoting the exposure of CD4i epitopes on gp120 [43,44]. Indeed, A32 Abs were found to bind in greater quantity to the surface of CD4+ T lymphocytes infected with Nef and Vpu-deficient HIV-1 compared to wild type-infected counterparts, with the degree of binding correlating with NK cell-mediated lysis of infected cells [43]. Furthermore, A32 Ab binding and subsequent ADCC was dependent on the presence of CD4 and the ability of the viral receptor to bind gp120 in Nef and Vpu-deficient HIV-1-infected target T cells, suggesting that Nef and Vpu-mediated CD4 downregulation protects infected T cells against ADCC by preventing the exposure of CD4i epitopes on gp120.

Although both proteins reduce surface expression of CD4, Nef and Vpu act through distinct mechanisms. Nef is produced early in infection and is therefore able to affect CD4 surface levels immediately by targeting molecules already present on the cell membrane. Nef binds CD4 at the PM through its aa 57-WLE-59 and several other residues comprising a hydrophobic pocket [45,46]. Recruitment of AP-2 by Nef induces endocytosis of CD4 in a clathrin-dependent manner and ultimately targets the viral receptor to lysosomes for degradation [47]. AP-2 association requires both the di-leucine motif at aa 160-ExxxLL-165 of Nef and a second di-acidic motif found at aa 174-(D/E)(D/E)-175 [48]. The trafficking of CD4-containing vesicles to lysosomes is still under investigation, but seems to proceed through Nef interaction with β-COP [17,49], possibly along the endosomal sorting complexes required for transport (ESCRT) pathway [50].

In contrast to Nef, Vpu acts later in the virus life cycle by targeting newly synthesized CD4 molecules, causing their retention in the endoplasmic reticulum (ER) and subsequent delivery to the ER-associated degradation (ERAD) pathway [27,51,52,53,54]. Vpu-mediated CD4 degradation therefore ensures that CD4 surface levels are not replenished by newly synthesized molecules. The mechanism of CD4 downregulation by Vpu requires the physical interaction of the cytosolic domains of both proteins [55,56] and the recruitment of the SCF^β−TrCP1/2^ E3 ubiquitin ligase complex through the phosphorylated S52,56 motif of Vpu [21]. This results in lysine and serine/threonine-dependent poly-ubiquitination of the CD4 cytosolic tail [51,52,53]. Poly-ubiquitination of CD4 promotes the recruitment of the VCP-UFD1L-NPL4 dislocase complex, a late stage component of the ERAD pathway, which mediates the extraction of CD4 from the ER membrane to the cytosol [52,53]. Subsequent delivery of dislocated CD4 molecules to the proteasome results in their efficient degradation [51,52,53]. Therefore, by usurping the endocytosis and ERAD machineries, Nef and Vpu deplete CD4 at the surface of infected T cells, negating the detrimental effects of the viral receptor on cell viability and virus release, while preventing the exposure of ADCC-favored CD4i epitopes in gp120. 

## 5. Membrane-Associated Restriction Factors: BST2 and SERINC3/5

### 5.1. BST2, a Host Restriction Factor Counteracted by Vpu

#### 5.1.1. Inhibition of Virus Particle Release 

**Figure 2:**
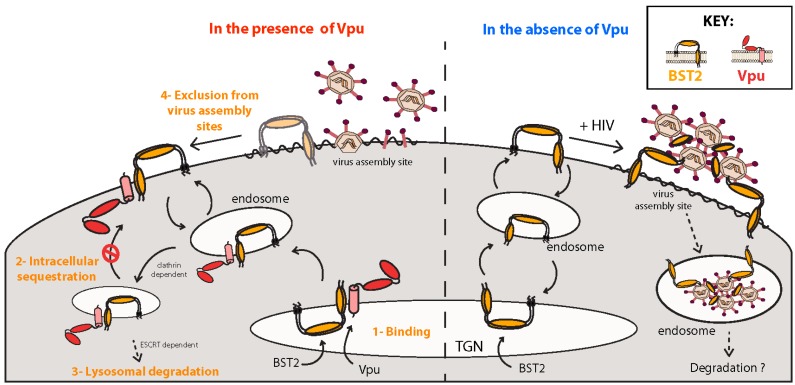
Multiple modes of Vpu-mediated bone marrow stromal cell antigen 2 (BST2) counteraction. Under normal conditions, *de novo* BST2 protein is supplied to the plasma membrane (PM) through the secretory system. Constitutive recycling of BST2 occurs from the PM to endosomes, as well as between endosomes and the *trans*-Golgi network (TGN) (**right**). During HIV-1 infection in the absence of Vpu, accumulation of BST2 at sites of viral assembly at the cell surface results in the incorporation of BST2 molecules into nascent virions, leading to inhibition of virus particle release. In select systems, these BST2-entrapped virions may be internalized and potentially degraded. During infection with Vpu-competent HIV-1, Vpu intercepts and binds BST2 within the vesicular system, resulting in their sequestration in endosomal structures in an AP-1 and clathrin-dependent manner (**left**). In the context of group M Vpu variants, sequestered BST2 proceeds to subsequent lysosomal degradation along the endosomal sorting complexes required for transport (ESCRT) pathway. Vpu can also bind BST2 at the cell membrane to induce its removal from sites of virus assembly using an AP-2 and clathrin-dependent system. This pool of BST2 may subsequently recycle into endosomal compartments for sequestration and degradation.

It was first observed over 20 years ago that Vpu-deficient HIV-1 viruses from the pandemic M group are impaired in their ability to release newly formed virions from the PM of infected T cells and macrophages [57]. Instead, progeny virus particles remain associated with the cell surface and accumulate in endosomal compartments [58] (Figure 2). This impairment of virus particle release was subsequently reported to be cell type and species specific [58,59,60], and it was later shown that type I interferon (IFN-I) could induce this restriction in permissive cells [61]. Electron microscopy studies have demonstrated that under restrictive conditions HIV-1 virions assemble properly and undergo maturation, yet remain “tethered” to the cell membrane. Based on these findings it was postulated that HIV-1 Vpu must counteract a yet-to-be-identified host factor capable of trapping virions to the cell membrane, and the search to identify this molecule began.

Two groups identified bone marrow stromal cell antigen 2 (BST2) (also designated Tetherin, CD317, HM1.24) as the IFN-I-inducible tethering molecule responsible for HIV-1 retention at the surface of infected cells [62,63]. BST2 is a type II integral membrane glycoprotein expressed as a homodimer at the cell surface. This protein is composed of a short N-terminal cytosolic tail that contains a conserved dual tyrosine-based trafficking signal (YDYCRV), a single pass α-helical TM domain, and a predominantly α-helical extended luminal region that ends in a membrane-embedded glycosylphosphatidylinositol (GPI) anchor at its C-terminus [64]. Dimerization appears stabilized by disulfide linkages and covalent interactions between the extracellular α-helices result in the formation of a coiled-coil structure [65,66]

BST2 dimers prevent the budding of HIV particles by incorporating one of their membrane-associated regions into the viral lipid envelope, preferentially the GPI anchor [67], while the other membrane-associated region remains within the cell membrane [68,69]. As expected, BST2 mutants lacking either the TM domain or GPI anchor are unable to tether particles [62,63,68]. The unique membrane topology of BST2, rather than its primary sequence, confers the virion tethering capability, as a chimeric molecule comprised of structurally similar motifs, yet sharing no sequence homology with BST2, is also able to prevent the release of HIV-1 virions [68]. It is interesting to note that physical retention of virions by BST2 often results in endocytosis of virus particles from the cell surface and their accumulation in endosomal compartments where they likely undergo degradation [63] (Figure 2). While this process is not required for the restriction of virus release *per se*, it may contribute to other antiviral functions of BST2 (see Section 5.1.3 below).

#### 5.1.2. Mechanisms of Vpu-Mediated BST2 Antagonism

The exact mechanisms by which Vpu antagonizes BST2 are still under investigation, but current knowledge indicates that Vpu interacts with BST2 to prevent its incorporation into budding virions. Vpu-mediated antagonism is associated with a reduction of BST2 levels at the PM, the site of its virion tethering activity, although Vpu-mediated antagonism has been noted in the absence of BST2 downregulation [70] (Figure 2). Vpu and BST2 interact directly through their respective TM domains and this association is essential for the counteraction of virion tethering [71,72]. This interaction occurs through residues A10, A14, A18 and W22 of Vpu, which form a hydrophobic ridge within the TM α-helix [73,74].

One distinctive feature of Vpus from group M HIV-1 is their ability to target BST2 to an ESCRT-dependent endosomal degradation pathway [75] through a process that is strictly dependent on the conserved β-TrCP-binding motif (S52,56) [76,77]. Recruitment of the β-TrCP-2 subunit of the SCF^β−TrCP1/2^ E3 ubiquitin ligase leads to direct ubiquitination of BST2’s cytosolic tail and degradation of the restriction factor in lysosomes [78,79] (Figure 2). However, recruitment of SCF^β−TrCP^ and the ensuing BST2 degradation are dissociable from Vpu’s ability to counteract virion retention [71,77,80,81,82,83]. Current evidence suggests that BST2 antagonism by Vpu results primarily from a subversion of BST2 trafficking. This occurs prior to subsequent β-TrCP-dependent BST2 ubiquitination and lysosomal degradation. The acquired ability of Vpu variants from group M HIV-1 to degrade BST2 has been proposed to reflect the need to suppress other consequences of BST2-mediated virion restriction that might further impede viral fitness *in vivo* (see Section 5.1.3 below) [83,84,85].

Vpu has been shown to block newly synthesized and/or recycling BST2 from trafficking to the cell surface [81,86]. This disruption of BST2 trafficking is dependent on an acidic di-leucine sorting motif (aa 59-ExxxLV-64) located in the second α-helix of the cytoplasmic domain of most HIV-1 group M Vpu variants [82] (Figure 1B). Since Vpu-mediated BST2 antagonism is a strictly clathrin-dependent process [82,87], and recent structural and biochemical data have revealed that the ExxxLV motif can bind AP-1 [88], a unified model of BST2 mistrafficking by Vpu has emerged. Briefly, this model proposes that Vpu hijacks the AP-1-dependent membrane trafficking pathway to force BST2/Vpu complexes into clathrin-rich domains of endosomes and the TGN where these complexes undergo β-TrCP-dependent ubiquitination before subsequent lysosomal degradation (Figure 2). Hence, by acting as a connector between BST2 and AP-1, Vpu appears to commit BST2 to a trafficking pathway that is incompatible with its transit to the PM. Perhaps surprisingly, recruitment of AP adaptors to the ExxxLV motif of Vpu was also found to be regulated by the conserved aa 51-DSGxxS-56 motif. The DSGxxS motif therefore appears to play two independent roles in BST2 antagonism: regulation of AP interactions with the ExxxLV motif for clathrin-mediated trafficking, and recruitment of β-TrCP for BST2 ubiquitination and targeting for lysosomal degradation [83]. In agreement with this, CK-II-mediated phosphorylation of S52,56 of Vpu, but not β-TrCP recruitment, was recently found to regulate recruitment of both AP-1 and AP-2 to the ExxxLV trafficking motif, implicating S52,56-dependent clathrin-based sorting as an essential first step in BST2 counteraction [83]. More interestingly still, given that AP-2 acts exclusively at the PM, the observation that AP-2 can also be recruited to the ExxxLV sorting motif raises the possibility that Vpu can also counteract BST2 by targeting the restriction factor to clathrin-rich domains of the PM, inducing its movement away from virus assembly sites. Consistent with such a scenario, fusion of Vpu’s cytosolic tail to BST2 was found to displace the restriction factor from sites of viral assembly at the PM in an ExxxLV motif-dependent manner [72]. This alternate mechanism of BST2 antagonism perhaps provides a rationale as to why in several lymphocytic cell lines, Vpu can counteract BST2 without downregulating it from the cell surface [70]. The ability of Vpu to hijack AP-dependent trafficking pathways suggests that it can affect BST2 transport/localization by several mechanisms (Figure 2). Importantly, it also describes a potential common theme in the mechanisms used by Vpu to downregulate various host surface proteins.

#### 5.1.3. Sensor of Virus Assembly and Activator of NFκB-Dependent Proinflammatory Responses

BST2 itself can also act as an innate immune sensor capable of inducing nuclear factor kappa-light-chain-enhancer of activated B cells (NFκB)-dependent proinflammatory responses from infected cells upon retroviral tethering at the PM [84]. Mechanistically, retention of virus particles induces clustering of BST2 dimers and phosphorylation of conserved tyrosine residues in their cytosolic tails, a condition that induces the recruitment of the spleen tyrosine kinase (Syk), which is required for downstream NFκB activation [89]. By preventing BST2-mediated viral retention, Vpu effectively limits activation of the NFκB pathway and subsequent release of proinflammatory mediators which would otherwise favor the detection of infected cells by the immune system.

The direct activation of NFκB signaling and restriction of virus particle release may not be the only way by which BST2 exerts its antiviral effects. Endocytic uptake of virions by BST2 and their subsequent degradation could potentially enhance the detection of viral components by endosomal pattern recognition receptors, such as Toll-like receptors (TLRs), and trigger the production of IFN-I [84] (Figure 2). Additionally, since Vpu-deficient virions accumulate in endosomal compartments within infected cells, it is conceivable that the BST2-mediated degradation of tethered virions during infection of antigen presenting cells (APC) (*i.e.*, macrophages) might enhance presentation of viral epitopes to augment T cell-mediated antiviral responses. Whether these ancillary effects of BST2-mediated virion tethering contribute to the overall antiviral activity of BST2 *in vivo* remains to be demonstrated. 

Translation beginning at alternative initiation sites generates two isoforms of BST2 with distinct biological activities. These isoforms differ only in the presence or absence of 12 aa at the cytosolic N-terminus and are capable of forming homodimers and heterodimers with each other [90]. The short isoform of BST2, which does not contain a conserved dual tyrosine-based motif found in the long isoform, is deficient for NFκB activation and is significantly more resistant to surface downregulation and degradation mediated by group M HIV-1 Vpu [85,90]. Notably, in most primate species BST2 clustering does not activate NFκB, presumably because of the molecular context around the conserved tyrosine residues [84,91]. Interestingly, the differential targeting (downregulation and degradation) of the long and short BST2 isoforms is not achieved by lentiviruses infecting primate species devoid of BST2-mediated NFκB activation [85]. The differential targeting of BST2 isoforms by Vpu from pandemic HIV-1 group M might therefore indicate that overcoming the human BST2 NFκB signaling capacity (long isoform) while still maintaining a pool of BST2 at the cell surface (short isoform) represented a critical adaptation required for the efficient zoonotic transmission that led to the HIV/AIDS pandemic.

#### 5.1.4. Distorting IFN-I Homeostasis through Altered Membrane-Initiated Signaling Cascades

Plasmacytoid dendritic cells (pDCs) are one of the two principal DC subsets found in humans. Amazingly, although pDCs constitute less than 1% of human peripheral mononuclear blood cells (PBMCs), they are the primary source of IFN-I produced during antiviral responses [92]. pDCs express CD4 as well as CXCR4 and CCR5, and as such support HIV-1 entry, particularly in the context of cell-to-cell contacts between infected T cells and pDCs, allowing for efficient viral sensing [93]. Detection of HIV-1 by pDCs is primarily mediated through recognition of single-stranded RNA by TLR7. Upon HIV-1 sensing, pDCs undergo activation which leads to the transcriptional upregulation of IFN-I and pro-inflammatory cytokines [94,95]. Interestingly, BST2 has been reported to be the ligand of immunoglobulin-like transcript 7 (ILT7), an inhibitory receptor on pDCs, whose activation and signaling suppress TLR7 and TLR9-mediated responses upon exposure to viral nucleic acids [96]. 

Recent data indicates that HIV-1 exploits this interaction to suppress IFN-I release by pDCs [97]. Tethering of HIV-1 particles to the PM of infected T cells was found to sterically occlude BST2 from interacting with ILT7 on pDCs, resulting in very efficient production of IFN-I by pDCs upon co-culture with T cells infected with Vpu-defective HIV-1. In contrast, expression of Vpu in HIV-1-producing cells downregulates most surface BST2, yet maintains a pool of BST2 outside viral assembly sites that is free to bind and activate ILT7 on pDCs. In this fashion, IFN-I production by pDCs is significantly suppressed in an ILT7-dependent manner. Current evidence suggests that the pool of BST2 persisting outside of viral assembly sites in the presence of Vpu is composed predominantly of the short isoform of BST2 [97]. Indeed, although not downregulated and degraded by Vpu, the short BST2 isoform is efficiently excluded from viral assembly sites (perhaps through Vpu/AP-2 interactions), and as such is still capable of interacting and activating ILT7. Therefore, through a highly sophisticated differential regulation of BST2 isoforms at the surface of infected cells, group M HIV-1 Vpu appears able to prevent virus particle retention and NFκB activation, while still attenuating IFN-I production by pDCs through ILT7 activation.

#### 5.1.5. Vpu, BST2 and ADCC

As discussed above, expression of BST2 at the surface of infected cells prevents the release of Vpu-deficient virions by tethering them to the PM. This generates a pool of viral antigens at the surface of infected cells, a condition that could enhance Ab opsonization, thus rendering infected cells susceptible to clearance by phagocytes and NK cells though Fc receptor recognition, including ADCC. Indeed, studies using various anti-HIV-1 Abs, including those found in HIV-1-infected patient sera, have shown that BST2-mediated virion tethering increases binding of ADCC-competent Abs to the surface of infected cells, increasing their ADCC-mediated deletion. Therefore, by counteracting BST2, Vpu decreases the quantity of viral Env epitopes found at the PM, resulting in lower NK cell-mediated ADCC activity directed against HIV-1-infected cells [43,98,99]. Interestingly, BST2-mediated virion tethering appears to work in concert with CD4i exposure of gp120 epitopes preferred by ADCC, such as that recognized by the A32 Ab (discussed above). In this context, HIV-1 suppresses ADCC activity through two distinct mechanisms: CD4 downregulation via Nef and Vpu, and BST2 antagonism via Vpu alone [43]. 

### 5.2. SERINC3/5, Host Restriction Factors Counteracted by Nef

Nef was first shown to promote HIV-1 infectivity over 20 years ago [100]. This effect has been observed in many *in vitro* model systems with Nef providing the greatest infectivity advantage in lymphoid cell lines and primary T cells. In addition, *nef* alleles taken from HIV-infected patients at different stages of infection all maintain a strong ability to promote virion infectivity [101], suggesting that this effect plays an important role *in vivo*. 

To increase viral infectivity Nef must be present in virus-producing cells, and inhibiting clathrin-mediated endocytosis in these cells abrogates this effect [102,103]. These observations led to the hypothesis that Nef alters the surface expression and incorporation of an unidentified “infectivity factor” into virus particles. Nef-induced infectivity enhancement was not observed when virions were pseudotyped with vesicular stomatitis virus glycoprotein G (VSV-G) or Rous sarcoma virus subgroup A (RSV-A) Env glycoprotein [104,105] but was maintained when viruses were pseudotyped with the amphotropic murine leukemia virus (A-MLV) Env glycoprotein [106]. Both VSV-G and RSV-A Env require endocytosis and endosome acidification to promote membrane fusion, while HIV and A-MLV Env can initiate fusion at neutral pH [107], suggesting Nef is able to overcome a hurdle to entry found at the cell membrane of target cells, but absent from endosomal structures. 

Recently the missing factor affected by Nef was identified independently by two groups [108,109]. The Pizzato group looked at global transcriptome profiles of several cell lines with varying degrees of Nef-induced infectivity enhancement in an attempt to correlate gene expression with this effect. The Göttlinger group used mass spectrometry to identify proteins that are differentially incorporated into virions in the presence or absence of Nef. Both groups identified serine incorporator (SERINC)3 and SERINC5 proteins as host factors that are excluded from budding HIV-1 virions in a Nef-dependent manner. Overexpression of SERINC3/5 in virus-producing cells in the absence of Nef dramatically decreases virion infectivity, resulting in a block at an early stage of infection, prior to reverse transcription, with SERINC5 providing greater restriction. Conversely, knockdown or deletion of these proteins greatly increases virion infectivity. Both Pizzato and Göttlinger groups also noted that virion entry does not appear to be inhibited prior to HIV-1 envelope fusion with the target cell membrane. Instead, there appears to be a block to infection at the fusion pore expansion step, perhaps the most energy costly stage in viral entry [110]. Furthermore, SERINC3/5 expression does not affect the infectivity of VSV-G pseudotyped viruses, in agreement with earlier data. Although further study is required, it has already been shown that Nef promotes the relocalization of SERINC5 to Rab7+ endosomal compartments in Jurkat T cells, perhaps suggesting that Nef targets these molecules for lysosomal degradation [108]. 

## 6. Tetraspanins: Virus Assembly Areas and the Control of Membrane Fusion

The tetraspanin family of proteins is involved in the formation of surface membrane microstructures referred to as tetraspanin-enriched microdomains (TEMs). TEMs act as protein scaffolds similar to lipid rafts, although these two structures are biochemically distinguishable, with TEMs being more soluble in strong detergents [111]. HIV-1 assembly occurs selectively at TEMs on the cell membrane [112,113], likely due to Gag/tetraspanin interactions [114]. Not surprisingly TEMs are also predominant at the virological synapse (VS). Similar in many ways to immunological synapse (IS) structures, VS structures are cell-to-cell contacts formed between infected and target cells that facilitate cell-to-cell HIV-1 transmission [112] and are believed to be responsible for the majority of HIV-1 spread *in vivo*. 

Tetraspanins also inhibit the fusion of lipid membranes through a poorly characterized mechanism [115,116]. In the context of HIV-1, fusion events resulting from interactions between Env expressed at the surface of infected cells and CD4 expressed on neighboring cells results in the formation of multinucleated syncytia *in vitro* and *in vivo* [117]. HIV Env-mediated membrane fusion must therefore be tightly regulated *in vivo*: fusion occurring prior to virion release may result in cell-to-cell fusion rather than viral spread, while fusion that is too prohibited may lower virion infectivity. Tetraspanins inhibit Env-mediated cell-to-cell fusion [118,119], with drug and molecular dye experiments suggesting a block at the hemi-fusion state [115]. Perhaps as an evolutionary compromise, tetraspanins are also packaged into HIV-1 virions where they generally lower particle infectivity by inhibiting fusion of viral envelopes with target cell membranes [120].

Nef and Vpu work in concert to downregulate surface expression of many tetraspanins on lymphoid cell lines including CD81, CD63, and CD53, amongst others [121,122]. Similar to numerous other Nef targets, this effect cannot be localized to any specific region within the Nef protein. Vpu-mediated downregulation of tetraspanins is independent of the TM sequence of Vpu and at least partially independent of the S52,56 motif, suggesting that Vpu targets these surface molecules without the need for specific binding within the membrane or recruitment of E3 ubiquitin ligase machinery. Surprisingly however, Nef and Vpu were shown to co-localize with tetraspanins in the TGN of 293T cells by confocal microscopy, and tetraspanins can be detected in Nef or Vpu co-immunoprecipitations (co-IPs) [122]. Furthermore, use of either lysosomal degradation inhibitors (bafilomycin A) or proteasome inhibitors (MG132) was found to prevent Vpu-mediated degradation of tetraspanins [121]. Hence, Vpu appears to target tetraspanins for downregulation and degradation by a novel mode of action not shared with canonical targets of Vpu (*i.e.*, CD4 and BST2).

With regards to TEMs, it is notable that recent studies have uncovered an unprecedented number of surface molecules downregulated by both Nef and Vpu [122,123]. In one study, 32 of 105 surface receptors analyzed were shown to be downregulated by both of these viral proteins [122]. Furthermore, similar to tetraspanins, many of the newly identified targets of Nef/Vpu-mediated downregulation do not require any previously described structural motifs of either Nef or Vpu (see below). The known role of tetraspanins in membrane microstructure formation therefore leaves open the possibility that many proteins downregulated by Nef and Vpu are not done so directly, but are rather targeted indirectly as a part of a larger disruption of TEM microstructures. For example, tetraspanins may be targeted by Nef and Vpu resulting in the downregulation of TEM-associated proteins by proxy, or *vice versa*. Caution must therefore be used when identifying novel cell surface targets of Nef and Vpu, as the potential for indirect targeting is high.

## 7. Inhibiting Immune Detection through Downregulation of Immunoreceptors: MHC-I/II, NKG2D-L, PVR, NTB-A, and CD1d

### 7.1. Nef Inhibits Adaptive Immune Responses: MHC-I/II

T cells recognize foreign antigens presented in the context of either major histocompatibility complex (MHC)-I or MHC-II molecules on the surface of infected cells. These interactions result in activation of adaptive immune responses, clonal expansion of antigen-specific lymphocytes and deletion of infected cells. By inducing the downregulation of MHC molecules, HIV-1 is able to prevent efficient adaptive immune responses, thus contributing to the persistence of infection.

**Figure 3:**
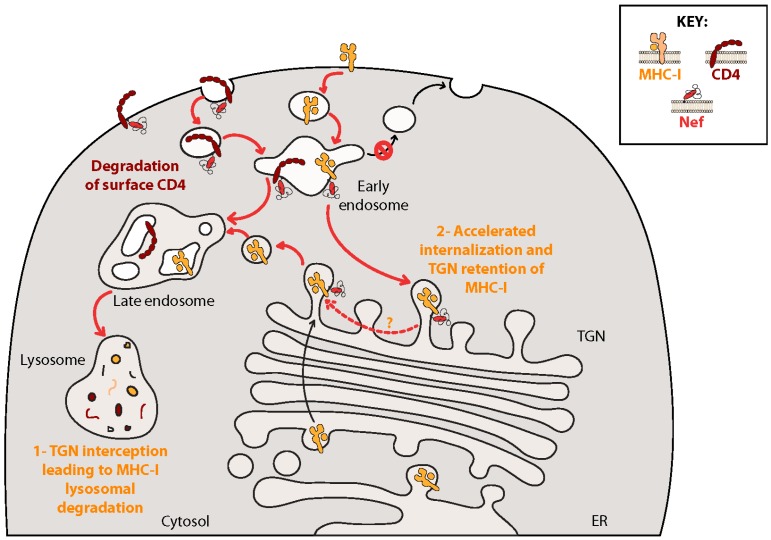
CD4 and major histocompatibility complex (MHC)-I are downregulated by HIV-1 Nef protein. Nef downregulates CD4 by recruiting the AP-2 adaptor to link CD4 to clathrin-mediated endocytosis, then targets CD4 for lysosomal degradation via β-COP-containing vesicles. Two mechanisms of Nef-mediated MHC-I downregulation have been proposed. Nef may bind *de novo* synthesized MHC-I in the TGN as a part of a Nef/AP-1/MHC-I trimolecular complex, eventually resulting in MHC-I lysosomal degradation, possibly along the same final vesicular pathway used for Nef-mediated CD4 degradation. A second model proposes that Nef induces the active removal of MHC-I from the plasma membrane (PM) by inducing a Srk family kinase (SFK)/phosphoinositide-3-kinase (PI-3K) signaling axis, and then prevents the homeostatic recycling of MHC-I molecules back to the cell surface. Instead, Nef induces retrograde transport of MHC-I to the TGN for sequestration. These mechanisms are not mutually exclusive and may occur in concert or at different time periods depending on the cell type.

Nef downregulates MHC-I [124] to protect infected cells from deletion by CTLs [125]. The mechanism of Nef-induced MHC-I downregulation remains in contention (Figure 3). It is clear that Nef affects MHC-I surface expression through a mechanism that is distinct from Nef-mediated CD4 downregulation, as these two effects require distinct regions of Nef. It is also established that binding of AP-1 to MHC-I is required for downregulation [126] and a crystal structure of a trimolecular complex formed between the AP-1 μ1 subunit, MHC-I and Nef has recently been solved [127]. Remarkably, the canonical AP-binding domain of Nef (aa 160-ExxxLL-165) does not appear to bind AP-1. Rather Nef binds to the AP-1 μ1 subunit through a hydrophobic pocket found at aa 13-WxxVxxxM-20 and the acidic cluster at aa 62-EEEE-65. This allows the aa 320-YSQAASS-326 sequence in the cytoplasmic tail of MHC-I to act as a substitute for another AP-1 binding motif (YxxΦ) [127,128]. 

Despite the known requirement of the AP-1/Nef/MHC-I trimolecular complex, controversy continues as to whether Nef acts primarily by blocking the anterograde transport of newly synthesized MHC-I molecules from the TGN to the cell surface, or whether Nef acts mostly by promoting the retrograde transport of MHC-I molecules from the cell surface to the TGN for retention (Figure 3).

In the first model, Nef blocks the anterograde transport of *de novo* synthesized MHC-I from the TGN to the cell surface by interacting with an immature hypophosphorylated form of the receptor [129]. MHC-I/AP-1/Nef ternary complexes are formed in the TGN and prevent trafficking of MHC-I towards the PM, instead targeting it for degradation though vesicles containing β-COP [129], possibly along the same final pathway as Nef-mediated CD4 degradation [49]. Indeed, short hairpin RNA knockdown of β-COP results in accumulation of MHC-I at the cell surface, prevents Nef-mediated downregulation and decreases co-localization of MHC-I with lysosomal markers [49], and Nef mutants unable to bind β-COP are unable to downregulate MHC-I [49,130].

A second, more complex, model proposes that Nef first traffics to the TGN through binding to PACS-2 where it interacts directly with various SFKs to induce their activation [131,132]. This initiates signaling through a phosphoinositide-3-kinase (PI-3K)-dependent cascade, ultimately promoting retrograde transport of MHC-I from the cell surface in adenosine diphosphate ribosylation factor (ARF)1 or ARF6-coated vesicles [131,132,133,134,135,136]. After removal from the cell surface, MHC-I is trafficked to endosomes containing both Nef and AP-1 where MHC-I recycling to the PM is inhibited. In this model MHC-I/AP-1/Nef complexes form in endosomal structures and are targeted to the TGN where MHC-I is retained by a mechanism dependent on Nef/PACS-1 interactions [137]. 

Difficulties coming to a consensus as to the exact mechanism of Nef-mediated MHC-I downregulation may indicate that Nef truly employs two or more methods to target MHC-I in a redundant fashion, or that different mechanisms are preferred in select situations and/or cell types. Indeed, using Nef inhibitors in primary CD4+ T cells, Dikeakos and colleagues demonstrated a temporally regulated switch in Nef’s mode of action, with Nef preferentially downregulating MHC-I from the cell surface for the first two days following infection, then primarily retarding transport of MHC-I from the ER to the PM at later time points [134].

Interestingly, the aa 320-YSQAASS-326 sequence of MHC-I essential for Nef/AP-1 complex formation is present in human leukocyte antigen (HLA)-A and HLA-B variants of MHC-I, but not in HLA-C and HLA-E [138,139]. HLA-A and HLA-B are primarily recognized by CTL-mediated immunity, while HLA-C and HLA-E act primarily as inhibitors of NK cell activation. This selective downregulation may promote the evasion of CTL immune surveillance while limiting NK cell-mediated cytotoxicity [139] (see below). 

With regards to MHC-II, Nef has been shown to increase expression of the invariant CD74 component at the cell surface of monocytic cell lines [140,141] and monocyte-derived macrophages (MDMs) from HIV-1 positive individuals [142]. Several groups have observed a concomitant downregulation of the mature MHC-II complex from the surface of infected cells [140,141,143]. Nef appears to actively remove MHC-II from the PM as well as prevent anterograde transport of MHC-II to the cell surface, shunting MHC-II towards lysosomal vesicles by a classic endocytic pathway involving Rab5+ early and Rab7+ late endosomes. This mechanism is semi-dependent on lipid raft formation yet completely independent of clathrin-mediated endocytosis [143].

### 7.2. Inhibiting Innate Immunity: Downregulation of NK Cell Activating Receptors

NK cells are cytotoxic lymphocytes that release cytolytic granules and immunomodulatory cytokines upon activation. The engagement of receptors expressed on the PM of NK cells conveys either inhibitory or activating signals into the cell [144]. MHC-I expression on target cells inhibits NK cell activation in response to pro-activating signals. Hence, by downregulating HLA-A and HLA-B molecules to avoid CTL deletion, HIV-1-infected cells may increase in susceptibility to NK cell-mediated killing. NK cells therefore act as a failsafe by interacting with infected cells and using the absence of T cell receptor (TCR)-stimulating signals as a cue to initiate a cytotoxic response. Importantly, NK cells are able to combat infection prior to the development of an adaptive immune response [145,146], and therefore these cells may have the potential to control the initial viral expansion at the port of entry prior to viral dissemination, leading to abortive infection. Avoiding destruction by NK cells during this critical early window (prior to full-fledged dissemination) may therefore be necessary for the establishment of systemic infection and viral reservoirs.

Both Nef and Vpu are known to target an ever-increasing number of molecules important for NK cell activation. Natural-killer group 2, member D (NKG2D) is an activating receptor found at the surface of NK cells and CD8+ T cells. Nef has been shown to downregulate numerous NKG2D ligands (NKG2D-L) from the surface of infected CD4+ T cells, resulting in the decreased cytolytic activity of co-cultured NK cell lines [147]. The mechanisms underlying this downregulation are not yet understood, yet a mutagenic analysis of Nef demonstrated that different regions of Nef are required for NKG2D-L and MHC-I downregulation. Nef also downregulates the ligand of another NK cell-activating receptor NKp44, to the same effect [148]. 

NK-T-B-antigen (NTB-A) is a type I TM glycoprotein self-ligand and member of the signaling lymphocytic activation molecule (SLAM) family of activating receptors expressed on all lymphocytes [149]. Engagement of NTB-A on NK cells provides a co-stimulatory signal to promote NK cell cytotoxic functions [149]. Vpu-mediated downregulation of NTB-A from the surface of infected CD4+ T cells results in decreased NK cell degranulation and specific lysis of HIV-1-infected T cells in co-culture experiments [150]. Unlike BST2 and CD4, Vpu does not alter whole cell levels of NTB-A. Consistent with this, NTB-A downregulation is independent of the β-TrCP-binding domain of Vpu (S52,56) and is not blocked by inhibition of SCF^β−TrCP^ [150,151]. Instead, Vpu appears to bind NTB-A through its TM domain and retain it in the TGN by preventing the conjugation and/or processing of NTB-A associated polysaccharides [150,152].

Polio virus receptor (PVR) is a ligand for the activating receptor DNAX accessory molecule-1 (DNAM-1) found on NK cells and CD8+ T cells [153]. Nef and Vpu downregulate PVR in an additive manner, resulting in decreased NK cell-mediated lysis of infected lymphoid cells [154]. Although the mechanism of Nef-mediated PVR degradation has not been thoroughly investigated, mutational investigations of Nef have mapped the effect to grossly the same motifs as required for MHC-I downregulation, suggesting a similar mechanism [154]. 

Vpu-mediated PVR downregulation is dependent on the same A10, A14, A18 TM sequence used to interact with BST2, yet does not require the C-terminal cytosolic ExxxLV motif required for BST2 downregulation nor the S52,56 motif, although reports on the requirement of S52,56 are somewhat conflicting [154,155]. Microscopy analysis suggests that, unlike CD4 or BST2, Vpu does not induce the degradation of PVR. Additionally, although microscopy images have revealed that Vpu co-localizes with PVR within perinuclear compartments, physical interaction between Vpu and PVR has not been detected, suggesting that the Vpu A10, A14, A18 TM ridge may bind an unknown intermediary to affect PVR expression, or that this ridge may be required for some other unknown function. 

Natural killer T (NKT) cells are T lymphocytes that share many features with NK cells and are found at anatomical sites of initial contact with microbes and allergens (e.g., epithelial mucosa). NKT cells express a semi-invariant TCR which does not recognize the classic MHC-I molecule, but rather binds to the MHC-like CD1d molecule loaded with microbial lipid antigens or stress-induced lipid self-antigens, resulting in NKT cell activation and release of both pro-Th1 and pro-Th2 cytokines [156]. In this manner NKT cells function as a bridge between innate and adaptive immune systems.

Vpu lowers surface expression of CD1d on productively infected monocyte-derived DCs resulting in lowered DC/NKT cell IS formation and decreased NKT cell activation [157,158]. Mechanistically, Vpu interferes with the homeostatic recycling of CD1d from endosomal compartments to the PM, trapping CD1d within early endosome antigen (EEA)-1+ early endosomal structures [157]. Interestingly, a Vpu mutant with a randomized TM domain was still able to downregulate CD1d, whereas a Vpu mutant completely lacking a TM region was unable to induce this effect [158]. This suggests that Vpu must be integrated into the membrane to act on CD1d expression, but does not interact specifically with the receptor through its TM domain. Like PVR and NTB-A, the S52,56 domain of Vpu is not required for CD1d downregulation [159], and Vpu does not induce CD1d degradation.

Similar to Vpu, Nef-mediated downregulation of CD1d also lowers NKT activation when co-cultured with HIV-1-infected DCs [160,161], although reports vary [130,162]. Nef binds directly to the C-terminus of CD1d, shown previously to be important for CD1d endocytosis under normal physiological conditions [163]. Similar to some other targets, Nef may retain CD1d in the TGN and actively increase its rate of endocytosis from the cell surface. Given the homology between MHC-I and CD1d [164], it is reasonable to hypothesize that Nef-mediated CD1d downregulation may occur through the same mechanisms as MHC-I downregulation. Surprisingly however, motifs of Nef important for both CD4 and MHC-I downregulation contribute to lowered CD1d expression to varying degrees [161], suggesting a distinct mode of action.

## 8. Altered Cell Homing: Downregulation of CD62L and CCR7

To properly combat microbial infection, lymphocytes must traffic between sites of infection and lymphoid organs through the blood and lymph systems. This homing behavior is achieved through a complex system of soluble chemokines and their corresponding surface receptors, as well as the expression of adhesion molecules at the cell surface capable of binding organ-specific counter-receptors. By altering the expression of these homing molecules (*i.e.*, chemokine receptors and adhesion molecules) on the surface of infected cells, HIV-1 can alter their trafficking behavior throughout the body, possibly contributing to disruption of immune functions. For example HIV-1 may inhibit migration of infected CD4+ T cells to peripheral lymphoid tissues to hinder the initiation of effective adaptive immune responses.

Recently HIV-1 Nef and Vpu were shown to downregulate the expression of two molecules important for the homing of lymphocytes into secondary lymphoid tissues: CCR7 [165] and CD62L [166]. CCR7 is a chemokine receptor that binds to the chemokines CCL19 and CCL21 produced by stromal cells within lymphoid structures, promoting the movement of lymphocytes towards these tissues [167]. Vpu has been shown to cause retention of CCR7 in the TGN through a mechanism dependent on the BST2-binding A14, A18, W22 TM hydrophobic ridge, resulting in decreased migration of primary human CD4+ T cells towards a CCL19 gradient [165]. Interestingly, although CCR7 downregulation does not occur in the presence of Vpu encoding mutations in the A14, A18, W22 ridge, co-IP experiments in 293T cells overexpressing Vpu and CCR7 have demonstrated that these proteins can physically associate independent of the composition of the Vpu TM region, thus suggesting that association of Vpu with CCR7 might be necessary but not sufficient to downregulate CCR7 from the PM of infected cells. In this context, the hydrophobic ridge may not directly interact with CCR7, but rather recruit additional cellular factors required for Vpu-mediated CCR7 downregulation. Unlike BST2 but similar to many of the more recently identified Vpu targets, CCR7 expression is not affected by the presence or absence of Vpu’s S52,56 motif and Vpu does not induce whole cell loss of CCR7, suggesting that Vpu does not degrade CCR7 through the SCF^β−TrCP^ E3 ubiquitin ligase system. Indeed, drug inhibition of SCF^β−TrCP^ does not affect Vpu-mediated CCR7 downregulation [151].

CD62L (L-selectin) is an adhesion molecule expressed on numerous leukocytic cell types. CD62L binds CD34 and glycosylation-dependent cell adhesion molecule 1 (GlyCAM-1) expressed on high endothelial venules, inducing both cell signaling and adhesion. This contributes to the “rolling and capture” behavior of lymphocytes, ultimately resulting in diapedesis of cells into secondary lymphoid organs [168]. Nef and Vpu downregulate CD62L on infected primary CD4+ T cells in an additive manner, resulting in impaired attachment of cells to fibronectin and decreased cell activation [166]. Both Nef and Vpu bind and retain CD62L in perinuclear spaces, presumably to prevent transport of the molecule to the cell surface. Nef reduces total cell CD62L levels without inducing receptor shedding via matrix metalloproteases, suggesting that Nef instead targets CD62L for degradation [166]. As is common with targets of Nef, CD62L co-localization and surface downregulation do not map entirely to any specific motif of Nef but are rather dispersed across numerous regions. With regards to Vpu, both co-localization of the viral protein with CD62L in perinuclear compartments as well as surface downregulation of CD62L are independent of the S52,56 motif and TM domain of Vpu [166]. Unlike Nef, Vpu does not reduce CD62L whole cell levels, consistent with a presumed lack of β-TrCP recruitment. 

## 9. Downregulation of Metabolic Transporters: SNAT1

A seminal work by Matheson and colleagues [123] has recently shown that HIV-1 group M Vpu downregulates a plethora of poorly characterized small molecule transporters from the surface of HIV-1-infected CD4+ T cells. These researchers focused on sodium-coupled neutral amino acid transporter (SNAT1), a small molecule transporter capable of carrying numerous metabolites across the cell membrane [169], with this study demonstrating a preference for alanine. Vpu targets SNAT1 for downregulation and lysosomal degradation using a SCF^β−TrCP^-dependent mechanism requiring both the TM aa A14 and W22 and the phosphorylated S52,56 motif of Vpu.

Importantly, Matheson and colleagues further showed that SNAT1 is poorly expressed in resting CD4+ T cells yet is dramatically upregulated at the cell surface following T cell activation. Correspondingly, CD4+ T cells drastically increase their alanine uptake in response to mitogenic stimuli in a SNAT1-dependent manner, thus promoting cellular proliferation. This corroborates previous evidence showing that T cell activation requires a large increase in amino acid metabolism [170]. By reducing SNAT1 levels at the PM, Vpu likely denies T cells the alanine required to undergo activation and activation-induced proliferation [170]. Downregulation of SNAT1 by Vpu may therefore represent a novel viral strategy to promote immune evasion. Indeed, decreasing the metabolic activity of infected cells by reducing alanine influx may decrease their production of viral epitopes, lowering detection of infected cells by adaptive immune responses. Inhibition of T cell activation in response to TCR stimulation may also contribute to the establishment of latent viral reservoirs by encouraging T cell quiescence, although this remains to be tested [171]. In light of its potential implications for viral persistence, the interference of HIV-1 Vpu with immunometabolism through decreased surface expression of SNAT1, and possibly other metabolites transporters, needs further study. 

SNAT1 downregulation is an activity that is well conserved among pandemic group M HIV-1 Vpu variants, and is found to a lesser extent in Vpus from related non-pandemic HIV-1 group N, and SIVcpz *Ptt* (the SIV variant that gave rise to group M HIV-1). In contrast, it is absent from Vpu variants from endemic HIV-1 groups O and P, as well as other more distantly related SIV lineages [123]. This indicates that Vpu-mediated SNAT1 downregulation is a biological activity most probably acquired recently in the evolutionary history of HIV. Whether SNAT1 downregulation contributes to the higher pathogenicity of group M HIV-1 viruses remains to be determined.

## 10. Conclusions

Overall, increasing evidence indicates that HIV-1 employs Nef and Vpu in a cooperative manner to dramatically alter the composition of the PM. As non-structural proteins, HIV-1 Nef and Vpu have evolved to increase viral fitness by altering the surface expression of important cellular proteins, including the viral receptor CD4, restriction factors, immunoreceptors, homing molecules, tetraspanins and membrane transporters (Table 1 and Table 2). By using both Nef and Vpu to target similar molecules at different cellular locations and times, HIV-1 has developed a remarkable ability to escape host immune responses. The division of labor between Nef and Vpu may have contributed to the extraordinary transmission fitness and virulence of the pandemic HIV-1 M subgroup as well as the initial zoonotic transmission of primate SIVs into human populations. This needs to be more thoroughly documented through a systematic analysis of Nef and Vpu variants from primate lentiviruses and other HIV-1 subgroups.

**Figure 4:**
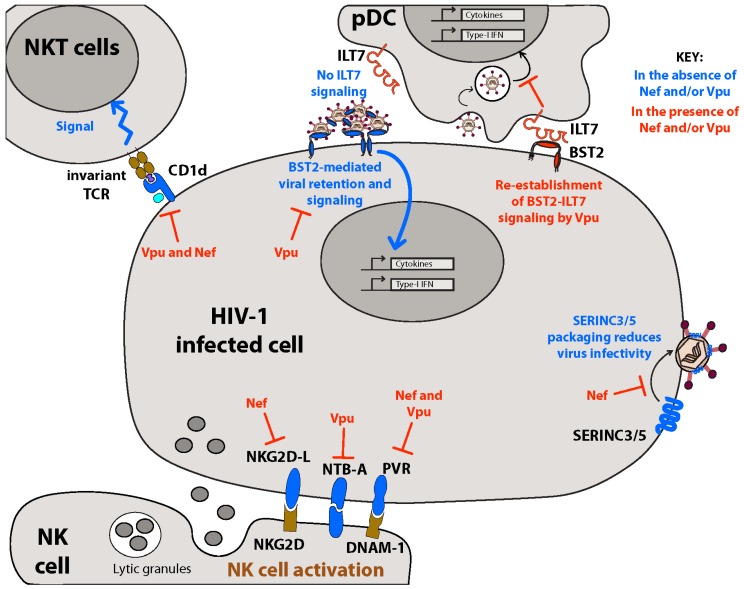
Remodeling of the plasma membrane by HIV-1 Nef and Vpu modulates intrinsic and innate immunity. HIV-1 Nef and Vpu proteins work in concert to alter the surface expression of host restriction factors (BST2 and SERINC3/5) and receptors/ligands important for innate immune responses. These processes promote viral immune evasion, allowing for dissemination and establishment of systemic spread during the acute phase of infection. Vpu-mediated selective regulation of the BST2 long and short isoforms at the cell surface prevents virion tethering at the PM, allowing efficient particle release while reducing NFκB activation in infected cells. Furthermore, differential targeting of the long and short isoforms of BST2 by Vpu allows the maintenance of a pool of surface BST2 that is free to engage and activate the plasmacytoid dendritic cells (pDC)-specific ligand of immunoglobulin-like transcript 7 (ILT7) inhibitory receptor, resulting in decreased type I interferon (IFN-I) production. Nef-mediated downregulation of serine incorporator (SERINC)3/5 decreases their packaging into virions and as such increases particle infectivity. Decreased surface expression of immunoreceptors such as natural-killer group 2, member D ligands (NKG2D-L), NK-T-B-Antigen (NTB-A), and polio virus receptor (PVR) by Nef and Vpu prevents clearance of infected cells by NK cells. Reduction of CD1d on infected DCs by Nef and Vpu prevents NKT cell activation and endorsement of immune responses.

Remodeling of the PM by Nef and Vpu is likely to have important biological implications during initial transmission events and subsequent persistent infection. By negating both intrinsic restriction factors (*i.e.*, BST2 and SERINCs) and innate immune responses (*i.e.*, pDC and NK(T) cell activity) during acute infection, Nef and Vpu are likely to promote both local replication at the site of initial infection and subsequent systemic spread (Figure 4). Indeed, counteracting intrinsic cellular restriction mechanisms is of highest priority to the virus, as these factors are inherent to most cells regardless of immune responses and therefore operate at the earliest stages of infection. Similarly, evading NK cell-mediated antiviral responses during acute infection is likely essential, as avoiding detection and clearance of infected cells is a pre-requisite for systemic viral dissemination and establishment of viral reservoirs. 

**Figure 5:**
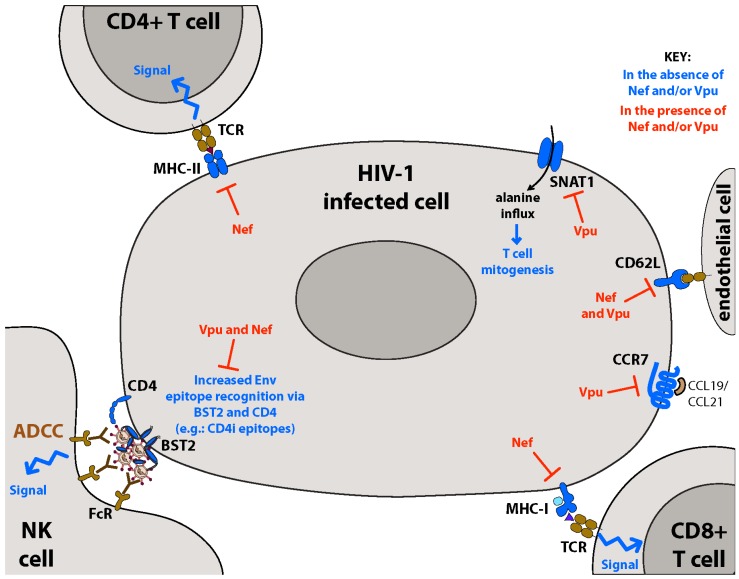
Remodeling of the plasma membrane by HIV-1 Nef and Vpu modulates immunometabolism and adaptive immunity. HIV-1 Nef and Vpu proteins work in concert to alter the expression of factors important for adaptive immune responses and optimal immunometabolic function, contributing to viral persistence during the later stages of HIV-1 acute infection. Downregulation of MHC-I on infected cells by Nef prevents their recognition and clearance by cytotoxic T lymphocyte (CTLs). Decreased surface expression of MHC-II on infected antigen-presenting cells (APCs) by Nef prevents optimal initiation of adaptive immune responses. Downregulation of both CD4 and BST2 by Nef and Vpu prevents the accumulation of CD4-induced (CD4i) gp120 epitopes at the cell surface, and therefore hampers anti-HIV antibody-dependent cell-mediated cytotoxicity (ADCC) responses. Reduction of sodium-coupled neutral amino acid transporter (SNAT)1 surface expression by Vpu reduces the intracellular alanine pool in infected CD4+ T cells, resulting in their decreased mitogenic capacity in response to TCR-activating stimuli. Downregulation of CD62L and CCR7 on infected cells may inhibit their trafficking to lymphoid structures, further decreasing the initiation of anti-HIV adaptive responses.

During the later stages of acute infection, HIV-1 persists despite robust adaptive immune responses. HIV-1 Nef and Vpu contribute to this persistence by disrupting adaptive immunity in a variety of ways (Figure 5). In addition to downregulation of MHC molecules, disruption of T cell metabolism through downregulation of SNAT1 (and possibly other metabolite transporters) may further hide infected cells from adaptive responses by inducing their quiescence, while dysregulation of cell homing through downregulation of CCR7 and CD62L may prevent the movement of infected cells to lymph nodes where adaptive responses are generated. Given the essential role of PM remodeling for initial infection, systemic dissemination, and viral persistence, thoroughly identifying all surface proteins modulated by Nef and Vpu, as well as the underlying molecular and cellular mechanisms, remain important research goals. Indeed, recent reports have revealed that Nef and Vpu modulate many more host surface proteins than previously believed, with the majority of these modulations having still undefined biological consequences [122,123]. Future studies in these areas will provide novel insight into HIV-1 pathogenesis while uncovering new avenues for therapeutic intervention.

New treatments designed to disrupt the function of Nef and Vpu will likely aid host immunity combat HIV-1 infection by restoring normal PM physiology. In fact, Nef and Vpu antagonists are currently in development [176,177]. Given the ability of Nef and Vpu to overcome intrinsic restriction factors and innate immunity, these drugs may have benefits as part of prophylactic regimens designed to prevent infection. Moreover, given the ability of Nef and Vpu to circumvent adaptive immune responses (CTL and ADCC), interfering with Nef and Vpu remodeling of the PM has the potential to increase the clearance of HIV-1-infected cells. Disrupting Nef and Vpu functions may therefore also provide therapeutic benefits as part of curative shock and kill strategies designed to stimulate HIV-1 gene expression from latent viral reservoirs while simultaneously promoting their elimination by anti-HIV-1 host adaptive immune responses.

## Figures and Tables

**Table 1:** Host PM proteins targeted by HIV-1 Nef.

Nef	Molecule Type	Downregulation					Binding		
		Observed with	Potential mechanism	Nef domains required	Experimental model (mechanism)	Ref	Nef domains required	Experimental model (binding)	Ref
**CD4**	Viral receptor/ immunoreceptor	Infection CD4+ T cells	Increased endocytosis via clathrin pathway. Lysosomal degradation.	160ExxxLL; 174DD; 154EE; 57WLE	Many model cell lines and methodologies	[172]	57WLE; G95, G96, L97, R106, L110	Direct *in vitro* binding with NMR	[45,46]
**SERINC3/5**	Intrinsic restriction factor	Infection CD4+ T cell	Relocalization to Rab7+ endosomes.	D123, 164LL	Trans. Exp. Jurkat	[108,109]	Not reported	Microscopy Trans. Exp. Jurkat	[108]
**Tetraspanins**	Membrane microstructure organizers	Infection CD4+ T cell	Intracellular sequestration in TGN.	Multiple (diffuse)	Trans. Exp. 293T	[121,122]	Not reported	CoIP Trans. Exp. 293T	[122]
**MHC-I**	Immunoreceptor	Infection CD4+ T cell	1. Increased endocytosis and sequestration. 2. Shunting of *de novo* MHC-I from TGN to lysosomes for degradation.	72PxxPxxP; 62EEEE; R17, R19; 13WxxVxxxM; W113, Y120	Many model cell lines and methodologies	[173]	Interacts in complex with AP-1 through 13WxxVxxxM; 62EEEE	X-ray crystallo-graphy	[127]
**MHC-II**	Immunoreceptor	Infection MDMs	Increased clathrin-independent, Rab-dependent endocytosis and decreased anterograde transport. Lysosomal degradation.	Multiple (diffuse)	Trans. Exp. U937/KO mouse BMDM	[143]	Not reported	Microscopy Trans. Exp. U937	[143]
**NKG2D-L**	Ligand of NK cell activating receptor	Infection Jurkat	Not reported	Multiple (diffuse)	N/A	[147]	N/A	N/A	N/A
**PVR**	Ligand of NK cell activating receptor	Infection CD4+ T cell	Intracellular sequestration in perinuclear areas without degradation.	72PxxPxxP; 62EEEE; F191	Trans. Exp. HeLa	[154,155]	Not reported	Microscopy Trans. Exp. HeLa	[155]
**CD1d**	MHC-like immunoreceptor	Infection Jurkat	Increased rate of internalization. Intracellular sequestration in the TGN.	Multiple (diffuse)	Infection Jurkat; Trans. Exp. 293T/HeLa	[160,161]	Not reported	CoIP Trans. Exp. T2	[160]
**CD62L**	Adhesion molecule/ homing	Infection CD4+ T cell	Intracellular sequestration. Decreased protein levels.	Multiple (diffuse)	Transd. Jurkat; Trans. Exp. 293T	[166]	Not reported	Microscopy Trans. Exp. 293T	[166]

Trans. Exp.: transient expression; Transd: transduction; Microscopy: co-localization by confocal microscopy; CoIP: co-immunoprecipitation; MDMs: macrophages; BMDM: bone marrow-derived monocytes; KO: Knockout.

**Table 2:** Host PM proteins targeted by HIV-1 Vpu.

Vpu	Molecule Type	Downregulation					Binding		
		Observed with	Potential mechanism	Vpu domains required	Experimental model (mechanism)	Ref	Vpu domains required	Experimental model (binding)	Ref
**CD4**	Viral receptor/ immunoreceptor	Infection CD4+ T cells and MDMs	Degradation via ERAD-like process.	TM; L63; V68; S52,56	Many model cell lines and methodologies	[53,172]	TM; cytosolic elements	CoIP Trans. Exp. HeLa; NMR	[174]
**BST2**	Intrinsic restriction factor	Infection CD4+ T cells and MDMs	Altered trafficking leading to intracellular sequestration and lysosomal degradation. Displacement from virus assembly sites.	TM; S52,56; 59ExxxLV	Many model cell lines and methodologies	[175]	TM (A10,14,18, W22)	CoIP Infection HeLa; NMR	[64,74]
**Tetraspanins**	Membrane microstructure organizers	Infection CD4+ T cells	Proteasome and lysosome-dependent degradation.	Undefined	Infection of lymphoid cells; Trans. Exp. 293T	[121,122]	Not reported	CoIP Trans. Exp. 293T	[122]
**NTB-A**	Co-stimulatory receptor. NK cell activating	Infection CD4+ T cells	Intracellular sequestration without degradation. Inhibition of NTB-A glycosylation.	TM (A18)	Trans. Exp. 293T/HeLa	[150,152]	TM	CoIP Trans. Exp. HeLa	[150]
**PVR**	Ligand of NK cell activating receptor	Infection CD4+ T cells	Intracellular sequestration without degradation.	TM (A10,14,18) S52,56?	Trans. Exp. HeLa	[154,155]	TM (A10,14,18)	Microscopy Trans. Exp. HeLa	[155]
**CD1d**	MHC-like immunoreceptor	Infection MDDCs	Intracellular sequestration in early endosomes without degradation.	Undefined	Trans. Exp. 293T	[157]	Not reported	CoIP Trans. Exp. 293T	[157]
**CCR7**	Homing receptor	Infection CD4+ T cells	Impaired recycling, and intracellular sequestration without degradation.	TM (A14,18, W22)	Trans. Exp. HeLa	[165]	Not reported	CoIP Trans. Exp. 293T	[165]
**CD62L**	Adhesion molecule/ homing	Infection CD4+ T cells	Intracellular sequestration without degradation.	Undefined	Trans. Exp. Jurkat/293T	[166]	Not reported	Microscopy Trans. Exp. 293T	[166]
**SNAT1**	Metabolite transporter	Infection CD4+ T cells	SCF^β-TrCP^-mediated ubiquitination for lysosomal degradation.	S52,56; TM (W22)	Stable Exp. Jurkat/HeLa	[123]	Not reported	CoIP Stable. Exp. HeLa	[123]

Trans. Exp.: transient expression; Stable Exp.: stable expression; Microscopy: co-localization by confocal microscopy; CoIP: co-immunoprecipitation; MDMs: macrophages; MDDCs: monocyte-derived dendritic cells.
